# p53 enhances elesclomol-Cu-induced cuproptosis in hepatocellular carcinoma via FDXR-mediated FDX1 upregulation

**DOI:** 10.3389/fonc.2025.1584811

**Published:** 2025-06-24

**Authors:** Xiao Liu, Honglin Qu, Jingmin Li, Xuhong Sun, Zhenlin Wang, Dong Wang, Xianyong Bai, Xiaoyan Li

**Affiliations:** ^1^ Department of Histology and Embryology, School of Basic Medical Sciences, Binzhou Medical University, Yantai, China; ^2^ Department of Human Anatomy, School of Basic Medical Sciences, Binzhou Medical University, Yantai, China; ^3^ Institute of Neurobiology, School of Basic Medical Sciences, Binzhou Medical University, Yantai, China; ^4^ Department of Introduction to Medicine, School of Basic Medical Sciences, Binzhou Medical University, Yantai, China

**Keywords:** p53, cuproptosis, ferredoxin reductase, elesclomol-Cu, ferredoxin 1, dihydrolipoyl transacetylase, hepatocellular carcinoma

## Abstract

**Background:**

Cuproptosis, a novel cell death pathway mediated by ferredoxin 1 (FDX1) and protein lipoylation, has emerged as a valuable target in cancer therapy. Although the findings of previous research have indicated a potential correlation between p53 and cuproptosis, the precise role and underlying mechanisms of p53 in cuproptosis, particularly within the context of hepatocellular carcinoma (HCC), remain unclear.

**Methods:**

To evaluate cuproptosis, three HCC cell lines (HepG2, PLC/PRF/5, and Hep3B2.1-7) with distinct p53 statuses were treated with elesclomol-Cu. p53 overexpression/knockdown, siRNA-mediated ferredoxin reductase (FDXR)/FDX1 knockdown, and the p53 activators CP-31398 and nutlin-3 were employed to elucidate the associated molecular mechanisms. Cell viability, protein expression [FDX1, dihydrolipoyl transacetylase (DLAT), FDXR], and DLAT oligomerization were assessed via Cell Counting Kit-8 (CCK-8), western blotting, and immunofluorescence analyses. A PLC/PRF/5 xenograft mouse model was used to assess combined the therapeutic efficacy of elesclomol-Cu and CP-31398.

**Results:**

Elesclomol-Cu triggered cuproptosis in HCC cells, as evidenced by a dose-dependent suppression of proliferation, FDX1 upregulation, DLAT oligomerization, and rescue by the copper chelator tetrathiomolybdate (TTM). p53 activation enhanced FDXR expression, promoting FDX1 upregulation and subsequent DLAT oligomerization, thereby sensitizing HCC cells to elesclomol-Cu, whereas FDXR knockdown reversed these effects, demonstrating its role in p53-mediated potentiation of cuproptosis sensitivity. In mutant p53-R249S cells, CP-31398 functioned synergistically with elesclomol-Cu to suppress proliferation. *In vivo*, elesclomol-Cu and CP-31398 combination therapy significantly reduced tumor growth and Ki67 expression whilst upregulating FDXR levels.

**Conclusions:**

These findings revealed that p53 enhances elesclomol-Cu-induced cuproptosis in HCC via FDXR-mediated FDX1 upregulation. This study provides mechanistic insights into p53’s role in cuproptosis and may serve as a basis for targeting copper metabolism in therapeutic strategies for HCC.

## Introduction

Liver cancer is the fourth leading cause of all cancer-related deaths globally, the incidence of which has increased in recent years in certain European and American countries (1, 2). Moreover, the annual incidence of new liver cancer cases has been predicted to increase by 55.0% between 2020 and 2040 (3). Hepatocellular carcinoma (HCC), which accounts for 75%–85% of all cases of liver cancer, is associated with a poor patient prognosis (4). Liver transplantation and tumor resection are the preferential treatments for patients diagnosed early, whereas therapeutic options for intermediate and advanced HCC, such as radiofrequency ablation and transarterial chemoembolization, often yield sub-optimal outcomes (5, 6). Moreover, whereas immune checkpoint inhibitors serve as first-line treatments for HCC, their efficacy tends to be limited to a small subset of patients (approximately 15%–30%) (7–9). Given the high incidence of HCC and limited efficacy of current therapeutic approaches, there is accordingly a pressing need to develop novel therapeutic strategies.

Tsvetkov and colleagues discovered that cell death induced by the copper ionophore elesclomol differs from other recognized forms of cell death, which led to the proposal of the term “cuproptosis” (10). Elesclomol transports copper to the mitochondria, wherein it interacts directly with lipoylated proteins such as dihydrolipoyl transacetylase (DLAT) in the tricarboxylic acid (TCA) cycle. This triggers the oligomerization of DLAT and induces instability in Fe-S cluster proteins, thereby eliciting proteotoxic stress, ultimately resulting in cell death (11), with ferredoxin 1 (FDX1) and protein lipoylation being identified as key regulators (12). The findings of recent studies have indicated that cuproptosis is linked to HCC progression, and, accordingly, genes associated with cuproptosis may serve as biomarkers for diagnosis and outcome prediction, and in guiding immunotherapy (13–15).

It has been established that cells dependent on mitochondrial respiration are almost 1,000 times more sensitive to elesclomol than those undergoing glycolysis, thereby revealing the close association between cuproptosis and mitochondrial metabolism (10). Given that p53 suppresses glycolysis and enhances oxidative phosphorylation in cancer cells via different mechanisms (16–18), it is plausible that this protein enhances cuproptosis by modulating metabolic shifts. p53 regulates the biosynthesis of the endogenous copper chelator glutathione (GSH) (19), and the depletion of GSH has been shown to promote cuproptosis (10). Additionally, genes involved in the biogenesis of Fe-S clusters, such as ferredoxin reductase (FDXR), which transfers electrons to FDX1, are regulated by p53 (20–22). Bioinformatic analyses have also revealed that TP53 mutation status is closely related to cuproptosis (23, 24). Collectively, the findings of these studies provide evidence to indicate a possible association between p53 and cuproptosis; however, the precise role and mechanisms of action of p53 in this context remain unclear.

In this study, using three HCC cell lines with distinct p53 statuses, we demonstrate that p53 enhances elesclomol-Cu-induced cuproptosis in HCC cells through FDXR-mediated FDX1 upregulation. Moreover, we found that combined treatment with elesclomol-Cu and a p53 activator markedly reduced HCC cell proliferation both *in vitro* and *in vivo*, thereby offering a promising treatment strategy for HCC.

## Materials and methods

### Cell culture, compounds and antibodies

The HCC cell lines Hep3B2.1-7 (p53 null) and PLC/PRF/5 (mutant p53 R249S) were obtained from the Cell Bank of the Chinese Academy of Sciences, and HepG2 cells (wild-type p53) were purchased from MeilunBio, Dalian, China. These cells were all cultured in minimum essential medium (PM150410; Procell, Wuhan, China) supplemented with 10% fetal bovine serum (Gibco, Thermo Fisher Scientific, USA) and 100 U/mL penicillin-streptomycin (PB180120; Procell, Wuhan, China) at 37°C in a 5% CO_2_ atmosphere.

Elesclomol (S1052) and nutlin-3(HY-50696) were purchased from Selleck Chemicals (Shanghai, China); CP-31398 (C287282) was purchased from Aladdin (Shanghai, China); the cell death inhibitors ferrostatin-1 (HY-100579; MedChemExpress), Necrostatin-1 (MB5067; MeilunBio, Dalian, China), *N*-acetyl-l-cysteine (MB1735; MeilunBio), and Z-VAD-FMK (HY-16658B; MedChemExpress) were obtained as indicated; and CuCl_2_ (751944) and tetrathiomolybdate TTM (323446) were purchased from Sigma–Aldrich (Germany).

Proteintech Technology (Wuhan, China) supplied the primary antibodies against Ki67 (27309-1-AP), FDXR (15584-1-AP), FDX1 (12592-1-AP), GAPDH (10494-1-AP), and p53 (60283-2-Ig), and DLAT antibodies were obtained from Cell Signaling Technology (#12362, USA).

### siRNA transfection

siRNAs targeting FDX1, p53, and FDXR were custom-synthesized by SANGON Biotech Co., Ltd (Shanghai, China). Cells were transfected with the corresponding siRNAs utilizing Lipofectamine 2000 Transfection Reagent (11668019; Thermo Fisher Scientific) according to the manufacturer’s instruction. Following incubation for 6 h, the culture medium was changed, followed by a further 24-h incubation. Subsequently, the cells were subjected to a 2-h pulse treatment using ES-Cu, followed by a further 24-h incubation. Scrambled siRNA: sense (5’-3’) UUCUCCGAACGUGUCACGUTT, antisense (5’-3’) ACGUGACACGUUCGGAGAATT. FDX1 SiRNA: 1# sense (5’-3’) CUAACAGACAGAUCACGGUTT, antisense (5’-3’) ACCGUGAUCUGUCUGUUAGTT; 2# sense (5’-3’) GUGAUUCUCUGCUAGAUGUTT, antisense (5’-3’) ACAUCUAGCAGAGAAUCACTT; 3# sense (5’-3’) UGGUGAAACAUUAACAACCAA, antisense (5’-3’) UUGGUUGUUAAUGUUUCACCA. FDXR SiRNA: 1# sense (5’-3’) GCUCAGCAGCAUUGGGUAU, antisense (5’-3’) AUACCCAAUGCUGCUGAGC; 2# sense (5’-3’) CACCAUUAAGGAGCUUCGG, antisense (5’-3’); CCGAAGCUCCUUAAUGGUG; 3# sense (5’-3’) GCUCAGCAGCAUUGGGUAUAA, antisense (5’-3’) UUAUACCCAAUGCUGCUGAGC. p53 SiRNA: 1# sense (5’-3’) CCCGGACGAUAUUGAACAATT, antisense (5’-3’) UUGUUCAAUAUCGUCCGGGTT; 2# sense (5’-3’) GACUCCAGUGGUAAUCUACTT, antisense (5’-3’) GUAGAUUACCACUGGAGUCTT.

### Overexpression of p53

HepG2 and Hep3B2.1–7 cells were plated in 6-well plates (4 × 10^5^ cells/well) and 96 well plates (5000 cells/well), and incubated until reaching 60%–80% confluence. Cells were transfected with 2.5 μg of the overexpression plasmid pCMV-TP53-3×FLAG-Neo (Miaoling Biotech, Wuhan, China) using 7.5 μL of Lipofectamine 2000 transfection reagent. The reagents used for transfection and subsequent cell-handling procedures were the same as those used for siRNA transfection.

### Cell viability assay

A CCK-8 assay kit (Beyotime Biotechnology, Shanghai, China) was used to assess cell viability. After seeding in 96-well plates (8000 cells/well), cells were treated with either CP-31398 (5 μg/mL) or TTM (20 μM) overnight or subjected to transfection. Subsequently, the cells were exposed to a 2-h pulse treatment using ES-Cu, followed by medium replacement and further incubation for 24 or 48 h. The subsequent steps were performed according to the manufacturer’s instructions.

### Evaluation of synergetic effect

The synergistic effects of CP-31398 and ES-Cu in PLC/PRF/5 cells were analyzed using Jin’s formula (25, 26). The cell proliferation inhibition rate was calculated as (A_control_ − A_treated_)/(A_control_ − A_blank_) × 100%. According to Jin’s formula *Q* = *Ea+b*/(*Ea* + *Eb* − *Ea* × *Eb*), where *Ea+b*, *Ea*, and *Eb* represent the mean inhibition rates of the combined treatment, CP-31398 alone, and ES-Cu alone, respectively, interactions were classified as antagonism (*Q* < 0.85), additive effects (0.85 ≤ *Q* < 1.15), or synergism (*Q* ≥ 1.15).

### Western blotting

The concentrations of proteins extracted from HCC cells were determined using a BCA protein assay kit (Beyotime, Shanghai, China). SDS-PAGE, membrane transfer, and blocking procedures were performed in accordance with standard protocols. Having added primary antibodies (anti-FDX1, anti-FDXR, anti-p53, and anti-DLAT, all diluted 1:1000; anti-GAPDH, dilution 1:20000), cells were incubated overnight at 4°C, and the following day were incubated with HRP-conjugated secondary antibodies (Proteintech, Wuhan, China). Subsequently, ECL luminescence reagent (Meilunbio, Dalian, China) was used for immunoblot imaging using a iBright CL750 Imaging System (Thermo Fisher Scientific). The levels of protein expression were standardized using GAPDH as an internal control and assessed using ImageJ software.

### Immunofluorescence staining

Cells were incubated for 30 min with a pre-warmed (37°C) working solution of 200 nM MitoTracker Red CMXRos (MB6046; Meilunbio, Dalian, China), followed by fixation with 4% paraformaldehyde. Primary antibody (anti-DLAT, dilution 1:100) and secondary antibody (Dylight 488-conjugated Goat Anti-Rabbit IgG, A23220; Abbkine, Wuhan, China, dilution 1:300) were sequentially added at appropriate times for incubation. Nuclei were counterstained with DAPI (Solarbio, Beijing, China). Finally, the slides were sealed with an antifade mounting medium (Meilunbio, Dalian, China) and visualized using a STELLARIS 5 confocal laser microscope (Leica, Germany).

### Mouse xenograft model

Four-week-old male BALB/c nude mice (Jinan Pengyue Biotechnology) were housed in a specific pathogen-free-grade animal facility at Binzhou Medical University. Subcutaneous tumor xenografts were established by injecting 5 × 10^6^ PLC/PRF/5 cells, dispersed in 100 µL PBS, into the left posterior flank (axillary region) of mice. Upon reaching a tumor volume of 100–150 mm^3^, mice were randomly allocated to one of three groups (five mice per group) and were accordingly intraperitoneally administered (1) PBS (control), (2) ES (10 mg/kg) + CuCl_2_ (0.06 mg/kg), or (3) CP-31398 (0.5 mg/mouse) + ES (10 mg/kg) + CuCl_2_ (0.06 mg/kg) on a daily basis. After five daily injections, the mice were given a 2-day rest interval before the administration cycle was repeated once more. Subsequently, tumor dimensions were measured, with volumes being calculated as (L × W^2^)/2 (L: length; W: width). All experimental protocols were approved by the Animal Ethics Committee of Binzhou Medical University.

### Immunohistochemistry

Tumor tissue sections (5 µm thick) were dewaxed and rehydrated using xylene and a graded series of ethanol, respectively. Antigen retrieval was thermally induced using citric acid buffer (pH 6.0) in a microwave oven, with exposure to high power for 4 min, followed by medium power for 16 min. Thereafter, the sections were subjected to endogenous peroxidase inactivation and serum blocking, followed by incubation with either an anti-FDXR antibody (dilution 1:200) or an anti-Ki67 antibody (dilution 1:8000). Secondary antibody incubation and chromogenic reactions were performed using a GTVision III Detection System/Mo&Rb (inclusive of DAB) (GeneTech, Shanghai, China), followed by hematoxylin counterstaining.

### Statistical analysis

*In vitro* experiments were performed independently three times. GraphPad Prism 9 software was used to analyze the data. Statistical analyses were performed using either the Student’s t-test or a one-way analysis of variance (ANOVA). Statistical significance was set at *P* < 0.05, and experimental data are reported as the means ± SD.

## Results

### Elesclomol-Cu induces cuproptosis in HCC cells

**Figure 1:**
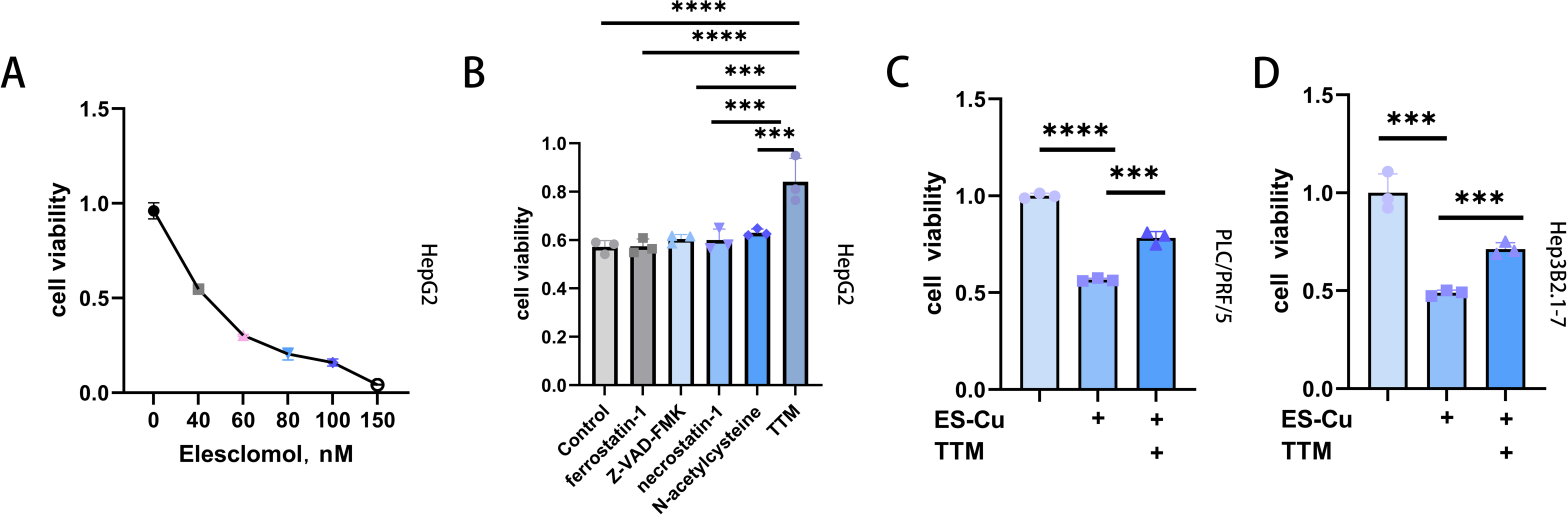
Elesclomol-Cu-induced cell death is distinct compared to various recognized forms of cell death. **(A)** Viability of HepG2 cells after pulse treated with various concentrations of elesclomol (ES) and 10 µM CuCl_2_ for 2 h and then cultured for an additional 48 h **(B)** Viability of HepG2 cells pretreated with ferrostatin-1 (10 μM), Z-VAD-FMK (30 μM), necrostatin-1 (20 μM), NAC (1 mM), TTM (20 μM) overnight and then pulse treated with ES (40 nM)-CuCl_2_ (10 μM). Control cells were treated with ES (40 nM) + CuCl_2_ (10 μM) alone. **(C, D)** Viability of PLC/PRF/5 **(C)** and Hep3B2.1-7 **(D)** cells pretreated with or without TTM, followed by pulse treatment with ES-Cu ((PLC/PRF/5: 40 nM ES + 10 μM CuCl_2_; Hep3B2.1-7: 30 nM ES + 10 μM CuCl_2_) for 2 h and then cultured for an additional 48 h. *** *p* < 0.001; **** *p* < 0.0001.

On the basis of previous findings indicating that elesclomol mediates copper-dependent cell death (27), in this study, we examined the effects of different concentrations of elesclomol (0, 40, 60, 80, 100, and 150 nM) combined with CuCl_2_ (10 µM) on the viability of HepG2 cells, and accordingly observed that elesclomol-Cu suppressed cell proliferation in a dose-dependent manner (**Figure 1A**). Whereas treatment with Z-VAD-FMK (an apoptosis inhibitor), *N*-acetylcysteine (NAC, an inhibitor of oxidative stress), ferrostatin-1 (a ferroptosis inhibitor), and necrostatin-1 (a necroptosis inhibitor) proved to be ineffective in rescuing cells from the cell death triggered by elesclomol-Cu (**Figure 1B**), the copper chelator TTM was found to cause a significant reversal of this effect, thereby indicating that elesclomol-Cu induces cuproptosis in HepG2 cells (**Figure 1B**). To further validate the effects of elesclomol-Cu, two further HCC cell lines (PLC/PRF/5 and Hep3B2.1-7) were treated with elesclomol-Cu for 2 h, the results of which revealed that elesclomol-Cu can also inhibit the viability of these HCC cells, and this effect could be reversed by TTM, consistent with its proposed role in inducing cuproptosis (**Figures 1C, D**).

**Figure 2:**
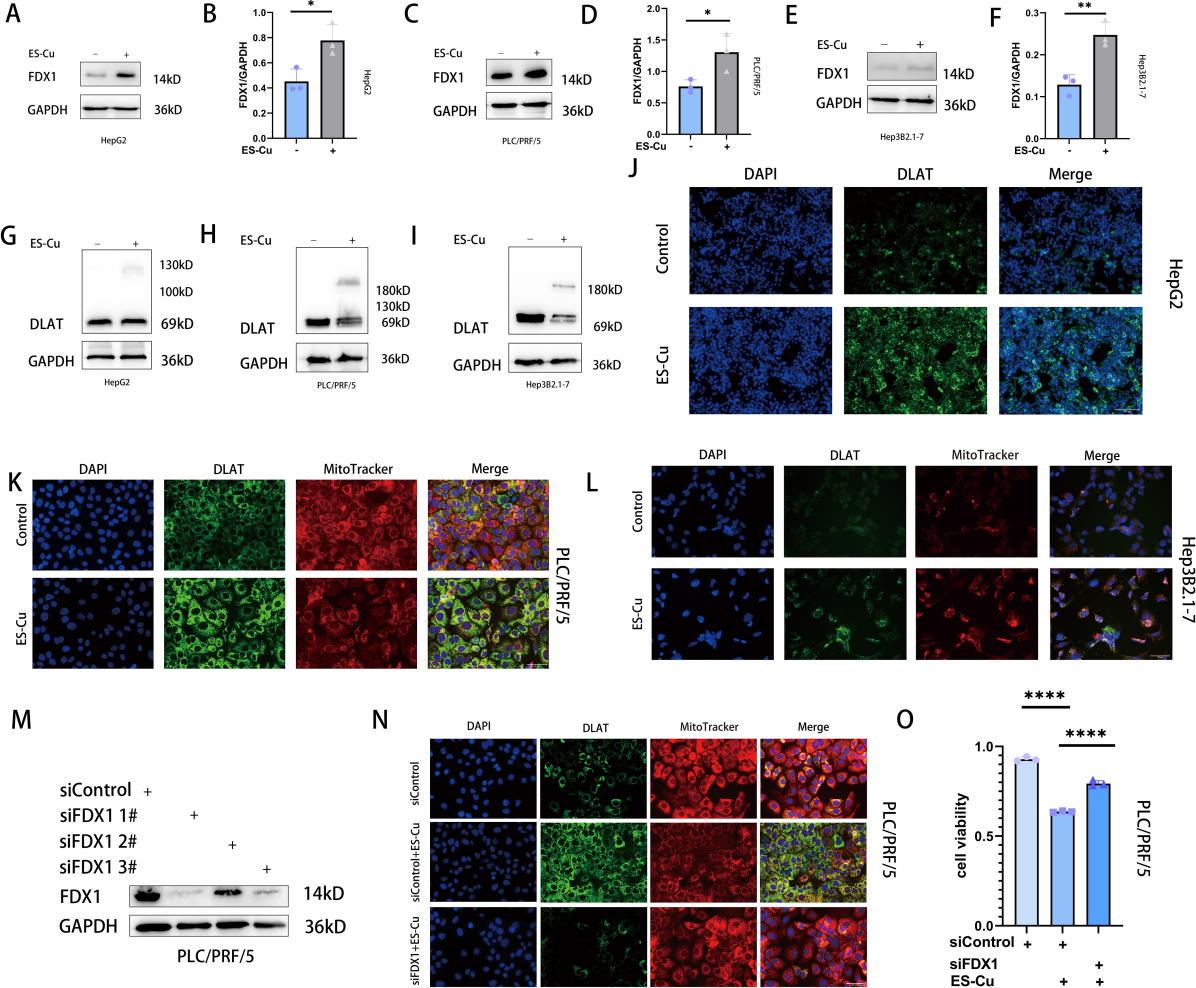
ES-Cu treatment upregulates FDX1 expression and promotes DLAT oligomerization. **(A-F)** Western blot analysis of FDX1 in HepG2, Hep3B2.1–7 and PLC/PRF/5 cells 24 h after 2h-pulse-treatment with ES-Cu. **(G-L)** Protein oligomerization was analyzed by western blot **(G-I)** and immunofluorescence imaging (**J-L**, DLAT-green, MitoTracker-red, DAPI-blue) in HepG2, Hep3B2.1–7 and PLC/PRF/5 cells 24 h after 2h-pulse-treatment with ES-Cu. **(M, N)** Protein oligomerization analyzed by immunofluorescence imaging **(N)** in FDX1 knockdown PLC/PRF/5 cells. **(O)** Viability of FDX1-knockdown PLC/PRF/5 cells after pulse treated with ES-Cu. * *p* < 0.05; ** *p* < 0.01; **** *p* < 0.0001.

We further examined the effects of treatment on the proteins FDX1 and DLAT, which are closely associated with cuproptosis, and accordingly observed a notable upregulation of FDX1 expression in all three assessed HCC cell lines following treatment with elesclomol-Cu (**Figures 2A-F**). Concurrently, DLAT oligomerization was promoted, thereby enhancing its toxic effects to a certain extent (10) (**Figures 2G-L**). Conversely, the knockdown of FDX1 was found to attenuate the effects of elesclomol-Cu (**Figures 2M, N**) and markedly reversed elesclomol-Cu-induced cell death (**Figure 2O**). These findings thus indicate that elesclomol-Cu induces cuproptosis in HCC cells, and that FDX1 plays a pivotal role in this process.

### p53 upregulates FDX1 expression and enhances elesclomol-Cu-induced cuproptosis

**Figure 3:**
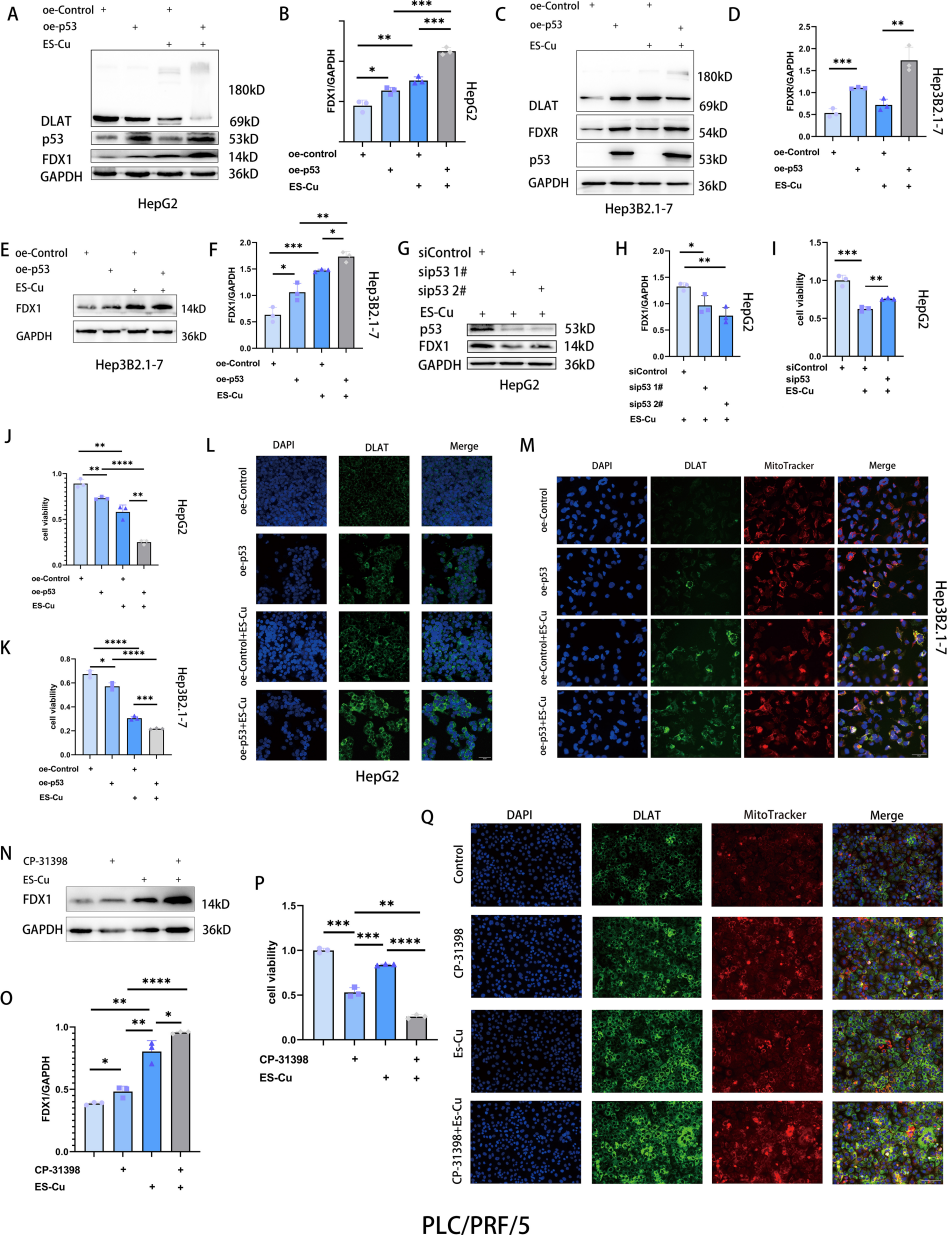
p53 upregulates FDX1 expression and enhances cuproptosis in HCC cells. **(A-F)** Western blot analysis of FDX1, FDXR and DLAT in p53-overexpressing HepG2 and Hep3B2.1–7 cells 24 h after 2h-pulse-treatment with ES-Cu. **(G, H)** Western blot analysis of FDX1 in p53-knockdown HepG2 cells with ES-Cu treatment. **(I)** Viability of p53-knockdown HepG2 cells 24 h following 2h-pulse-treatment with ES-Cu. **(J, K)** Viability of p53-overexpressing HepG2 **(J)** and Hep3B2.1–7 cells **(K)** after pulse treatment with ES-Cu. **(L, M)** Protein oligomerization was analyzed by immunofluorescence imaging in p53-overexpressing HepG2 **(L)** and Hep3B2.1–7 cells **(M)**. **(N, O)** Western blot analysis of FDX1 treated with ES-Cu, CP-31398 or a combination of both compounds in PLC/PRF/5 cells. **(P)** Viability of PLC/PRF/5 cells following exposure to ES-Cu, CP-31398 or a combination of both compounds. **(Q)** Protein oligomerization was analyzed by immunofluorescence imaging. * *p* < 0.05; ** *p* < 0.01; *** *p* < 0.001; **** *p* < 0.0001.

p53 acts as a potent tumor suppressor by regulating different types of cell death (28–31). To examine the association between p53 and cuproptosis, we overexpressed p53 in HepG2 cells, which was found to induce a significant suppression of cell viability (**Figure 3J**) and promoted the upregulated expression of FDX1 (**Figures 3A, B**). Conversely, p53 knockdown suppressed FDX1 expression and attenuated elesclomol-Cu-induced cell death (**Figures 3G-I**). Notably, combined treatment with overexpressed p53 and elesclomol-Cu markedly enhanced the expression of FDX1 and DLAT oligomerization compared with treatment with elesclomol-Cu alone (**Figures 3A, B, L**). Moreover, the combined treatment was found to have a more pronounced suppressive effect on cell proliferation than either p53 overexpression or elesclomol-Cu treatment applied individually (**Figure 3J**). Similar results were obtained using Hep3B2.1**–**7 cells, indicating that p53 upregulates FDX1 expression, thus enhancing elesclomol-Cu-induced cuproptosis in HCC cells (**Figures 3C-F, K, M**).

In liver cancer, *TP53* is the most commonly mutated gene, and p53-R249S is the only established hotspot mutation in HCC (32–34). Mutations in the TP53 gene often acquire novel oncogenic driver activity, termed gain-of-function (GOF). Specifically, the p53-R249S mutant achieves its GOF activity through methylation mediated by its upstream regulator SETDB1 and by enhancing c-Myc-dependent cell proliferation (33, 35). CP-31398, a p53 activator, has been shown to restore the DNA binding capacity of p53-R249S in a dose-dependent manner, thereby functionally rescuing mutant p53 to act similarly to wild-type p53 (36). To further examine the effects of p53 on cuproptosis in HCC cells, we treated PLC/PRF/5 cells (harboring the p53-R249S mutation) with CP-31398, and accordingly found that CP-31398 treatment upregulated FDX1 expression (**Figures 3N, O**). Compared with the elesclomol-Cu treatment alone, the combined treatment with CP-31398 and elesclomol-Cu promoted significant increases in FDX1 expression and a more substantial inhibitory effect on cell proliferation (**Figures 3N-P**). Using Jin’s formula, we found that the combination of CP-31398 and elesclomol-Cu exhibited synergistic effects in PLC/PRF/5 cells (Q = 1.17). Furthermore, immunofluorescence analysis revealed DLAT oligomerization (**Figure 3Q**). These findings thus indicate that restoration of p53 function enhances the cytotoxic effects of elesclomol-Cu, further corroborating the association between cuproptosis and p53.

### p53 targets FDXR to upregulate FDX1 expression and enhance cuproptosis sensitivity

**Figure 4:**
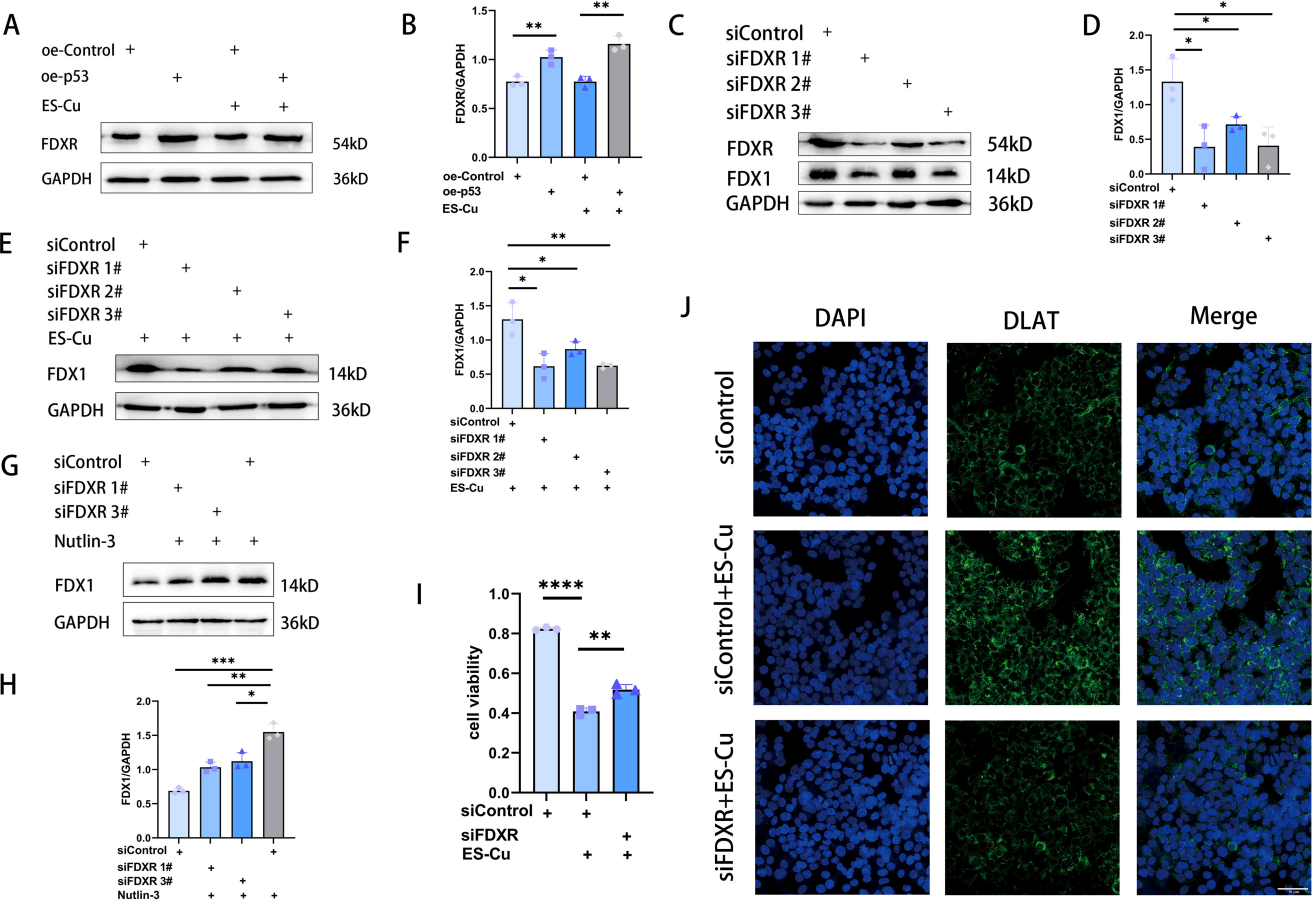
p53 targets FDXR to enhance cuproptosis in HCC cells. **(A, B)** Western blot analysis of FDXR in p53-overexpressing HepG2 cells 24 h after 2h-pulse-treatment with ES-Cu. **(C-F)** Western blot analysis of FDX1 in FDXR-knockdown HepG2 cells with or without ES-Cu treatment. **(G, H)** Western blot analysis of FDX1 in FDXR-knockdown HepG2 cells with nutlin-3 (10 μM) treatment. **(I)** Viability of FDXR-knockdown HepG2 cells 24 h following 2h-pulse-treatment with ES-Cu. **(J)** Protein oligomerization was analyzed by immunofluorescence imaging in FDXR-knockdown HepG2 cells. * *p* < 0.05; ** *p* < 0.01; *** *p* < 0.001; **** *p* < 0.0001.

FDXR is a mitochondrial-associated flavoprotein that transfers electrons from NADPH to FDX1 and FDX2, thereby influencing cellular processes such as Fe-S cluster biogenesis, steroid transformations, and protein lipoylation (37–39). Moreover, as a direct transcriptional target of p53 (21), a deficiency in FDXR is associated with liver disease and tumorigenesis (40, 41). In HCC cells overexpressing p53, we observed an upregulated expression of FDXR, thereby indicating that FDXR is directly targeted by p53 in these cells (**Figures 3C, D**; **4A, B**). As previously described, the overexpression of p53 leads to an upregulation of FDX1 expression, thereby enhancing elesclomol-Cu-induced cuproptosis in HCC cells. To investigate whether this process is mediated via the regulation of FDXR, we knocked down FDXR in HCC cells and, as a consequence, observed a concomitant reduction in FDX1 expression (**Figures 4C, D**). The same effect was also observed in HCC cells treated with elesclomol-Cu (**Figures 4E, F**). Furthermore, FDXR knockdown was found to inhibit elesclomol-Cu-induced DLAT oligomerization (**Figure 4J**), and partially attenuated elesclomol-Cu-induced cell death (**Figure 4I**). Nutlin-3, which induces p53 accumulation by inhibiting its interaction with MDM2 (42), was found to upregulate FDX1 expression, while knockdown of FDXR abolishes this nutlin-3-induced FDX1 upregulation (**Figures 4G, H**). These findings suggest that p53 enhances FDX1 expression via FDXR regulation, thereby increasing the sensitivity of HCC cells to elesclomol-Cu.

### Combination of p53 activator CP-31398 and elesclomol-Cu significantly suppresses tumor growth

**Figure 5:**
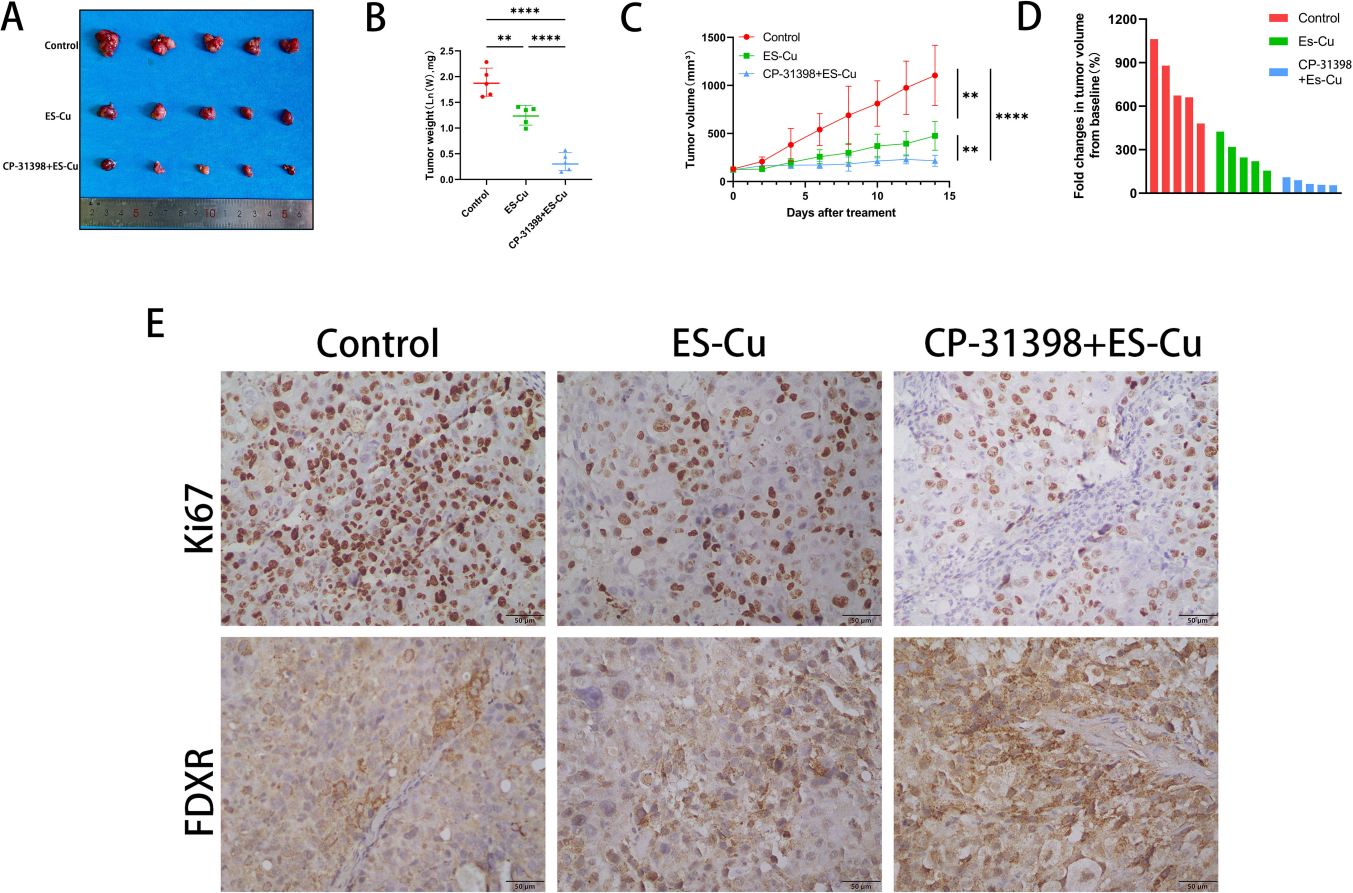
Combination of p53 activator CP-31398 and elesclomol-Cu significantly suppresses tumor growth. **(A)** Gross images of tumors at the end (14 days after treatment). **(B)** The weight of the resected tumors was measured in the control, ES-Cu, and ES-Cu+CP-31398 groups. **(C)** Graph of tumor volume changes in the control, ES-Cu, and ES-Cu+CP-31398 groups. **(D)** Tumor volume fold changes in mice treated with the indicated regimens. Each bar represents an individual mouse (n=5 per group). Calculation formula: fold change = ((V1-V2)/V2) * 100% (V1: tumor volume at endpoint; V2: tumor volume measured on the initial day of treatment). **(E)** Tumor samples were stained immunohistochemically for Ki67 and FDXR. ** *p* < 0.01; **** *p* < 0.0001.

The findings of our *in vitro* studies revealed that p53 enhances the sensitivity to elesclomol-Cu by upregulating the expression of FDXR in HCC cells. Moreover, the combined treatment with elesclomol-Cu and the p53 activator CP-31398, which targets the p53-R249S mutation, resulted in a more pronounced inhibitory effect on HCC cell proliferation. On the basis of these findings, we speculate that the combination of a p53 activator and elesclomol-Cu can suppress tumor growth *in vivo*. To validate this supposition, we established a PLC/PRF/5 nude mouse xenograft model, with the tumor-bearing mice being treated with PBS, elesclomol-Cu, or a combination of elesclomol-Cu and CP-31398. The findings revealed that elesclomol-Cu potently inhibited tumor growth, with the combined therapy having an even more pronounced antitumor efficacy (**Figures 5A-D**). These observations were further corroborated by immunohistochemical analysis, which revealed a notable reduction in Ki67 staining in the tissues of mice receiving the combined treatment compared with those treated with elesclomol-Cu alone (**Figure 5E**). Notably, we also observed a substantial elevation in the expression of FDXR within tumor cells subjected to the combined treatment (**Figure 5E**), thereby providing additional experimental support for the hypothesis that p53 enhances elesclomol-Cu-induced cuproptosis by upregulating the expression of FDXR.

## Discussion

The increasing incidence of HCC, coupled with the poor response of tumors to current therapeutic measures, emphasizes the urgency of developing novel treatment strategies (43). The copper ionophore elesclomol has long been considered an anticancer agent, primarily on account of its capacity to selectively induce the generation of mitochondrial reactive oxygen species (ROS), thus leading to oxidative stress (44, 45). The introduction of the concept of cuproptosis has, nevertheless, encouraged researchers to re-examine elesclomol from a new perspective. Elesclomol can bind to and transport Cu (II) to the mitochondria, wherein FDX1 reduces Cu (II) to Cu(I), which then binds directly to lipoylated DLAT, thereby promoting its oligomerization and ultimately inducing cell death via cuproptosis (10, 27, 46). The findings of our *in vitro* experiments revealed that a pulsed regimen of elesclomol and copper chloride effectively suppresses HCC cell proliferation in a dose-dependent manner. *In vivo* experiments further confirmed its notable tumor-suppressive effects, thus providing preliminary evidence for the application of this copper ionophore in HCC therapy.

Treatment of three different HCC cell lines with elesclomol-Cu in all cases revealed an upregulated expression of FDX1, which, by supplying electrons to cytochrome P450 proteins, has been established to regulate the metabolism of bile acids, vitamins, and steroid hormones (47, 48). Recent studies have revealed that FDX1 functions as an upstream modulator of protein lipoylation (10), and that its loss leads to a complete disruption of protein lipoylation and impairment of DLAT oligomerization, thereby conferring resistance to cuproptosis (10). However, the reported changes in FDX1 expression following elesclomol-Cu treatment have tended to be inconsistent (49–52), which can probably be ascribed to differences in cell type, cellular heterogeneity, and off-target effects at high elesclomol concentrations. FDX1 can function as an electron donor to initiate lipoylation mediated via lipoic acid synthetase (LIAS) (39), or directly bind to LIAS, thereby promoting its interaction with lipoyl carrier proteins, and thus enhancing cellular protein lipoylation (53). Upregulation of FDX1 expression enhances lipoylation of DLAT, thereby facilitating the binding of Cu(I) to DLAT, inducing its oligomerization, and subsequently promoting cuproptosis in HCC cells, which could represent the mechanism underlying the improved prognosis observed in patients with HCC characterized by high FDX1 expression (15, 23, 54). Our cellular experiments in the present study further confirmed that the knockdown of FDX1 not only reduced elesclomol-Cu-induced DLAT oligomerization but also conferred resistance to copper-induced cell death in HCC cells.

Previous studies have shown that tumor cells with high FDX1 expression exhibit increased more susceptibility to cuproptosis under equivalent copper concentrations (54). In this study, we found that either p53 overexpression or treatment with p53 activators (CP-31398 and nutlin-3) promotes FDX1 expression. Concurrently, p53 overexpression or CP-31398 treatment enhanced the cytotoxicity of elesclomol-Cu, suggesting this effect is associated with FDX1 upregulation, while conversely, p53 knockdown suppressed FDX1 expression and alleviated elesclomol-Cu-induced cuproptosis. To further investigate the underlying mechanism by which p53 upregulates FDX1, we demonstrated that the overexpression of p53 increased FDXR expression, which in turn elevated FDX1 levels. Notably, knockdown of FDXR inhibited elesclomol-Cu-induced FDX1 expression and DLAT oligomerization, and attenuated elesclomol-Cu-triggered cell death. Based on these findings, we propose that p53 promotes elesclomol-Cu-induced cuproptosis in HCC cells via FDXR-mediated upregulation of FDX1, potentially representing a novel mechanism whereby p53 regulates cuproptosis.

*In vivo* studies demonstrated that CP-31398, a p53-R249S mutant activator, combined with elesclomol-Cu to suppress PLC/PRF/5 tumor growth. This antitumor effect was accompanied by elevated expression of FDXR, consistent with our previously proposed hypothesis. Given that the p53-R249S mutation accounts for approximately 26% of all reported p53 mutations in HCC (55, 56), the combined administration of CP-31398 and elesclomol-Cu represents a promising therapeutic approach for the treatment of HCC.

In conclusion, our findings reveal that p53 enhances elesclomol-Cu-induced cuproptosis in HCC cells by upregulating FDX1 expression via FDXR regulation. We also demonstrated the potential efficacy of combining p53 activators with elesclomol-Cu to significantly suppress tumor growth, thereby providing a novel therapeutic strategy with potential clinical applications in the treatment of HCC.

However, the absence of a CP-31398 monotherapy group in the xenograft model precludes rigorous demonstration of true pharmacological synergy ***in vivo***, despite ***in vitro*** synergy analyses suggesting cooperative effects between CP-31398 and elesclomol-Cu. At the same time, we fully acknowledge the preliminary nature and limited scale of these findings, and that the functions of p53 in tumor growth are multifaceted and sometimes have contradictory effects (19, 57, 58). Additionally, cuproptosis has also been established to be associated with other forms of cell death (59, 60). Consequently, further studies are required to comprehensively elucidate the association between p53 expression and cuproptosis.

## Data Availability

The original contributions presented in the study are included in the article/supplementary material. Further inquiries can be directed to the corresponding authors.
